# Transcriptome Analysis and Hub Gene Identification in the Brain Cell Lines of the Spotted Knifejaw (*Oplegnathus punctatus*) After Poly (I:C) Stimulation

**DOI:** 10.3390/ijms27021101

**Published:** 2026-01-22

**Authors:** Ruiqi Guo, Kaimin Li, Jinfeng Liu, Songlin Chen, Lei Wang

**Affiliations:** 1 State Key Laboratory of Mariculture Biobreeding and Sustainable Goods, Yellow Sea Fisheries Research Institute, Chinese Academy of Fishery Sciences, Qingdao 266071, China; 2 Laboratory for Marine Fisheries Science and Food Production Processes, Qingdao Marine Science and Technology Center, Qingdao 266237, China; 3 Shandong Provincial Key Laboratory of Animal Resistance Biology, College of Life Sciences, Shandong Normal University, Jinan 250014, China

**Keywords:** *Oplegnathus punctatus*, nervous necrosis virus (NNV), transcriptome, hub genes, aquaculture industry

## Abstract

The spotted knifejaw (*Oplegnathus punctatus*) has emerged as a species with substantial potential for aquaculture development in China. However, its industrial cultivation is severely constrained by viral diseases. Among these, viral nervous necrosis (VNN), caused by nervous necrosis virus (NNV), represents a critical bottleneck to the sustainable development of this industry. In order to elucidate the immune response mechanisms of the brain cells of spotted knifejaw, this study established a poly (I:C) stimulation model in vitro and performed transcriptomic sequencing to analyze the differentially expressed genes (DEGs) after stimulation. There were 3169, 3228, and 3262 DEGs at 3 h, 6 h, and 12 h compared to 0 h (control), respectively. Co-expression time clustering of DEGs identified two gene clusters (cluster 6 and cluster 10), which included several immune-related genes. GO and KEGG enrichment analyses indicated that DEGs among the four time points were significantly enriched in immune signaling pathways, including the NOD-like receptor, RIG-I-like receptor, C-type lectin receptor, and Toll-like receptor pathways, as well as disease-response pathways. In total, 1398 common DEGs were identified among three comparative groups, which delineated six interaction clusters and 30 hub genes in protein–protein interaction (PPI) network analysis. By integrating a cellular model with transcriptomics, this study provides preliminary insights into the molecular immune mechanisms underlying the response of brain cells to poly (I:C) stimulation, offering important theoretical support for future research on disease-resistant breeding and disease control strategies in spotted knifejaw.

## 1. Introduction

The spotted knifejaw *(Oplegnathus punctatus)*, a species belonging to the genus *Oplegnathus* within the family *Oplegnathidae* (order Perciformes), is mainly found in the coastal regions of China, Japan, and other Southeast Asian countries [1]. The spotted knifejaw is highly valued in the market for its tender flesh and delicate flavor. Its body exhibits an obtuse hexagonal shape with a grayish-brown coloration adorned with evenly distributed black spots. The body of the black spotted snapper exhibits an obtuse hexagonal profile, with a grayish-brown base coloration uniformly scattered with black spots. Due to the combination of silvery patterns and dark spots on the body surface, the fish displays an iridescent, dreamlike appearance under illumination, earning it the name “dreamfish” and making it a species of notable ornamental value [2]. With advances in artificial breeding technology and the development of relay aquaculture, spotted knifejaw has become an economically important species in China, widely cultured in industrial farming systems, net cages, and marine ranching operations [3].

Current research on spotted knifejaw has primarily focused on aquaculture biology, the origin of heteromorphic chromosomes, and gonadal development patterns [4]. Transcriptomic studies have shown that following infection with iridovirus or *Vibrio harveyi*, differentially expressed genes in the spleen of spotted knifejaw are significantly enriched in pathways such as the NOD-like receptor signaling pathway, Toll-like receptor signaling pathway, hematopoietic cell lineage pathway, and the cytosolic DNA-sensing pathway [5,6]. However, investigations into the disease resistance and immune mechanisms of spotted knifejaw brain cells remain limited. To address this gap, the present study aims to analyze transcriptomic data related to disease resistance and immunity in spotted knifejaw, thereby providing foundational insights into the immune system of this species.

Viral nervous necrosis (VNN), caused by nervous necrosis virus (NNV), a double-stranded RNA virus, affects more than 120 marine fish species due to its broad host range. NNV primarily targets the nervous system, inducing pathological changes, such as vacuolation in the brain and retina, which disrupt normal neural function and behavior. The mortality rates can reach 70–100% [7]. In infected spotted knifejaw, common clinical signs include body darkening, abdominal swelling, erratic swimming near the water surface, anorexia, and eventually death due to the cessation of nutrition intake despite the availability of feeding. Post-mortem examinations often reveal significant neural damage, including neuronal necrosis, degeneration, and cerebral edema, as well as hepatic and splenic abnormalities [8]. The virus is transmitted through both vertical and horizontal routes. Vertical transmission occurs when the virus is carried within the eggs of broodstock or attached to the surface of fertilized eggs, thereby passing from parents to offspring [9]. Horizontal transmission is facilitated by contaminated water, virus-carrying live feeds (e.g., rotifers, copepods), infected aquaculture equipment, and direct contact between fish [10]. To date, no commercial vaccine is available against this pathogen, which complicates disease control and poses a serious threat to a variety of marine fish species, including groupers, sea bass, and tongue sole.

Polyinosinic:polycytidylic Acid (poly (I:C)), a viral analogue, is widely used in both in vivo and in vitro studies to simulate viral infection. Research has demonstrated that poly (I:C) stimulation can significantly upregulate the expression of interferon-stimulated genes in various fish species [11]. For instance, intraperitoneal injection of poly (I:C) (0.1 mg/100 g fish) induced the expression of interferon regulatory factor 11 (IRF11), which subsequently amplified the antiviral signaling network by activating the promoters of IFNc, IFNd, and IFNh in yellowhead catfish *(Tachysurus fulvidraco*) and large yellow croaker (*Larimichthys crocea*) [12,13]. Similarly, injection of 2 mL of poly (I:C) led to a significant initial increase in leukocyte counts, serum complement levels, and interferon gene expression across multiple tissues at 3 h in Mandarin fish (*Siniperca chuatsi*), reflecting the rapid immune response post-viral challenge and providing insights for immune monitoring [14]. In this study, we used poly (I:C) to stimulate a brain cell line of spotted knifejaw to simulate NNV infection, with the aim of elucidating the antiviral response of spotted knifejaw to NNV and providing a theoretical basis for future disease control strategies.

## 2. Results

### 2.1. Quality Control of Transcriptome Sequencing and Sequence Alignment

To investigate the antiviral immune mechanisms in spotted knifejaw brain cells, transcriptome sequencing was performed following stimulation with poly (I:C), a synthetic viral RNA analog. Cells were harvested at multiple time points, and a total of 12 libraries were constructed, designated as infected0_1–3, infected3_1–3, infected6_1–3, and infected12_1–3. Sequencing was carried out using the DNBSEQ platform. On average, each library generated approximately 42.8 million clean reads and 6.4 Gb of clean bases, with Q20 and Q30 values exceeding 97% and 93%, respectively (Table 1).

### 2.2. Sample Correlation Analysis

**Figure 1:**
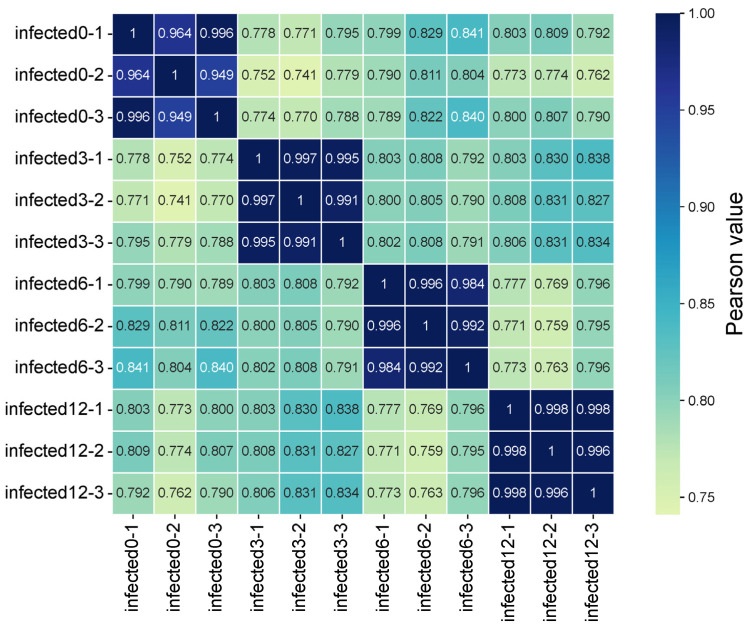
Pearson’s correlation heatmap of 12 spotted knifejaw brain cell samples. (Pearson values indicate pairwise comparisons between samples that quantify the linear relationship across different time points).

Sample reproducibility and sequencing data reliability were assessed by calculating correlation coefficients based on gene expression levels. Distinct clustering was observed between different treatment groups. The Pearson correlation coefficients between the 0 h group and three post-infection groups ranged from 0.770 to 0.841, while those among the three samples in same group ranged from 0.949 to 0.998, that indicating high reproducibility of transcriptome (Figure 1), indicating high reproducibility. These results confirm that all experimental procedures, including sample processing, RNA extraction, library construction, and sequencing, were performed consistently and reliably, supporting the validity of subsequent data analysis.

### 2.3. Analysis of Differentially Expressed Genes (DEGs)

**Figure 2:**
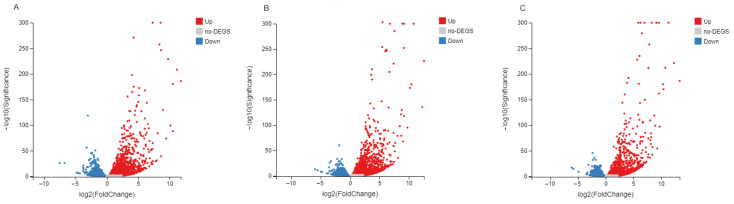
Volcano plots of differentially expressed genes (DEGs) in spotted knifejaw brain cells. (**A**) Comparison of 0 h vs. 3 h post poly (I:C) stimulation; (**B**) Comparison of 0 h vs. 6 h post poly (I:C) stimulation; (**C**) Comparison of 0 h vs. 12 h post poly (I:C) stimulation.

Gene expression profiles of poly(I:C)-stimulated spotted knifejaw brain cells were compared against the 0 h control at 3, 6, and 12 h post-stimulation (hps) to identify DEGs in each comparison. The analysis revealed 3169 DEGs at 3 hps (1818 up-regulated and 1351 down-regulated), 3228 DEGs at 6 hps (1942 up-regulated and 1286 down-regulated), and 3262 DEGs at 12 hps (1748 up-regulated and 1514 down-regulated) (Figure S1). Volcano plots further demonstrated distinct temporal patterns of gene expression changes, with variations in both the number and composition of DEGs across time points, reflecting the dynamic and time-dependent nature of the host transcriptional response to immune challenge (Figure 2).

### 2.4. Time-Series Expression Pattern Analysis of Differentially Expressed Genes

**Figure 3:**
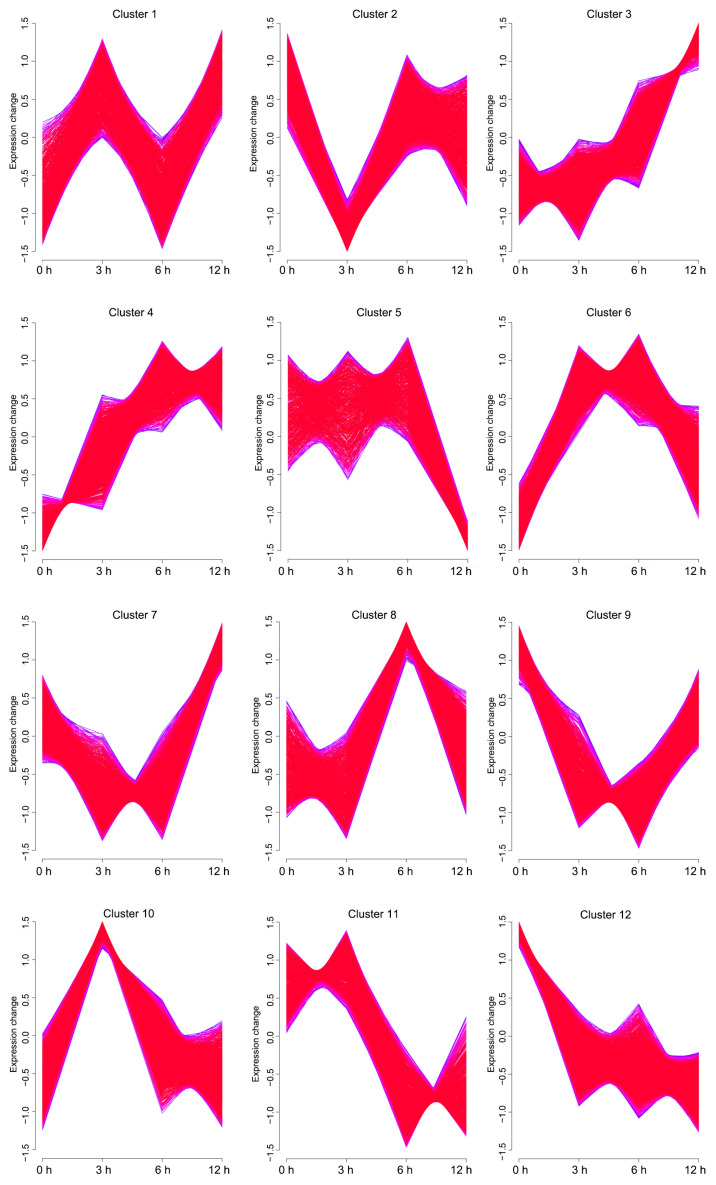
Time-series expression clustering of differentially expressed genes (DEGs) in spotted knifejaw brain cells following poly (I:C) stimulation. A total of 12 clusters were identified using the Mfuzz algorithm, and each cluster displays a unique temporal expression pattern.

According to the differences in the expression patterns of these DEGs, they were divided into 12 co-expression clusters. Analysis of temporal expression profiles revealed distinct response patterns among the gene clusters. Genes in clusters 1, 6, 10, and 11 exhibited significant upregulation at 3 h post-stimulation, indicating an immediate early response to poly(I:C) challenge. Clusters 2, 4, 5, 6, and 8 reached their expression peaks at 6 h, suggesting roles during the intermediate phase of immune activation. Meanwhile, clusters 1, 3, 4, and 7 showed peak expression at 12 h, implicating their functions in maintaining cellular responses during the late phase of stimulation (Figure 3).

### 2.5. Go and KEGG Pathway Enrichment Analyses of DEGs

**Figure 4:**
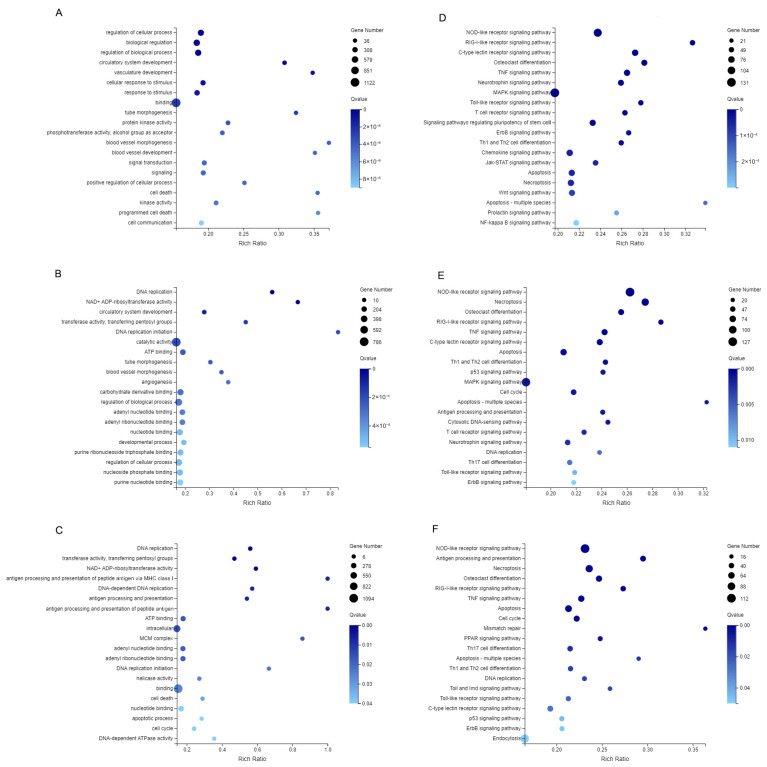
GO and KEGG pathway enrichment analyses of DEGs in spotted knifejaw brain cells. (**A**–**C**) GO enrichment results: (**A**) 0 h vs. 3 h, (**B**) 0 h vs. 6 h, (**C**) 0 h vs. 12 h. (**D**–**F**) KEGG pathway enrichment results: (**D**) 0 h vs. 3 h, (**E**) 0 h vs. 6 h, (**F**) 0 h vs. 12 h.

To elucidate the functional roles of DEGs following poly (I:C) stimulation, Gene Ontology (GO) enrichment analysis was performed on the DEG sets identified from the three pairwise comparisons (0 h vs. 3 h, 0 h vs. 6 h, and 0 h vs. 12 h). The analysis revealed significant enrichment in 122, 74, and 49 GO terms for the three comparison groups, respectively. GO enrichment analysis revealed distinct functional patterns among DEGs from different comparison groups. In the 0 h vs. 3 h group, DEGs were significantly enriched in broad regulatory processes, including “regulation of cellular process,” “biological regulation,” “regulation of biological process,” as well as response-related terms such as “cellular response to stimulus” and “response to stimulus”. In the 0 h vs. 6 h groups, DEGs were primarily associated with metabolic and catalytic activities, including “DNA replication,” “DNA replication initiation,” “NAD+ ADP-ribosyltransferase activity,” and “transferase activity, transferring pentosyl groups”. Then, the 0 h vs. 12 h group showed enrichment in immune-related processes, particularly “antigen processing and presentation of peptide antigen via MHC class I,” “antigen processing and presentation,” and “antigen processing and presentation of peptide antigen” (Figure 4A–C).

KEGG pathway enrichment analysis of the DEGs from each comparison group revealed significant enrichment of multiple immune-related pathways. In the 0 h vs. 3 h, 0 h vs. 6 h, and 0 h vs. 12 h groups, these included the NOD-like receptor signaling pathway, RIG-I-like receptor signaling pathway, C-type lectin receptor signaling pathway, and Toll-like receptor signaling pathway. Pathways associated with cell growth and death, such as Apoptosis and Necroptosis, along with signal transduction pathways like the TNF signaling pathway, were also significantly enriched. Additionally, the MAPK signaling pathway was prominently enriched in the 0 h vs. 3 h and 0 h vs. 6 h groups, while the p53 signaling pathway showed significant enrichment in the 0 h vs. 3 h and 0 h vs. 12 h groups (Figure 4D–F). Time-series genes in clusters 6 and 10 showed significant enrichment in immune-specific pathways such as the NOD-like receptor signaling pathway, RIG-I-like receptor signaling pathway, antigen processing and presentation, C-type lectin receptor signaling pathway, Toll-like receptor signaling pathway, Jak-STAT signaling pathway, TNF signaling pathway, and NF-kappa B signaling pathway (Figure S2).

### 2.6. Analysis of Co-Expressed DEGs Across Different Time Points

**Figure 5:**
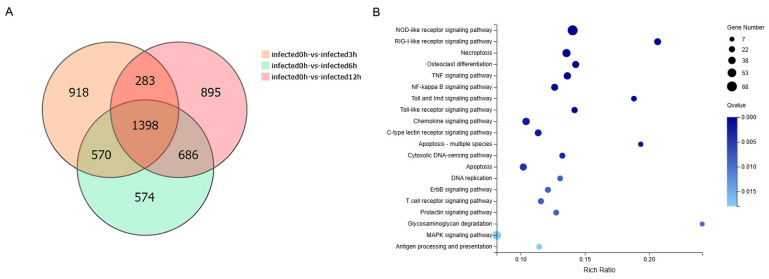
Venn diagram and KEGG bubble plot of common DEGs across different time points. (**A**) Venn diagram showing the overlap of DEGs among 0 h vs. 3 h, 0 h vs. 6 h, and 0 h vs. 12 h comparisons. A total of 1398 common DEGs were identified. (**B**) KEGG bubble plot of 1398 common DEGs. The *y*-axis represents the top 20 enriched KEGG pathways.

Venn analysis revealed distinct and overlapping DEG sets among the comparison groups. Specifically, 918, 574, and 895 genes were uniquely identified in the 0 h vs. 3 h, 0 h vs. 6 h, and 0 h vs. 12 h groups, respectively, while 1398 DEGs co-existed in all three comparisons (Figure 5A). KEGG enrichment analysis of these common DEGs identified 20 significantly enriched pathways. Based on bubble color intensity and enrichment score, the top five significantly enriched pathways were NOD-like receptor signaling pathway, RIG-I-like receptor signaling pathway, TNF signaling pathway, NF-kappa B signaling pathway, and Cytosolic DNA-sensing pathway (Figure 5B).

### 2.7. Protein–Protein Interaction (PPI) Analysis

**Figure 6:**
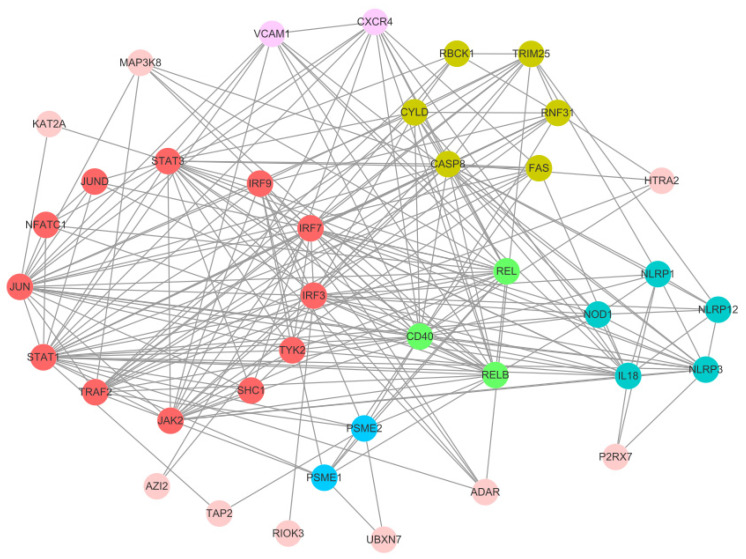
Protein–protein interaction (PPI) network of common DEGs in spotted knifejaw brain cells. Six functional clusters (Cluster 1–6) were identified via DBSCAN clustering, containing 30 hub genes.

From the 1398 common DEGs identified by Venn analysis, genes associated with the top 20 enriched KEGG pathways were selected for protein–protein interaction (PPI) analysis. The PPI network was constructed using the DBSCAN clustering algorithm in Cytoscape (v3.10.3). This analysis revealed six central functional clusters comprising 30 core genes: Cluster 1 included 12 genes (*STAT1*, *STAT3*, *IRF3*, *IRF7*, *IRF9*, *TRAF2*, *TYK2*, *SHC1*, *JAK2*, *JUN*, *NFATC1*, *JUND*), Cluster 2 contained 6 genes (*RBCK1*, *RNF31*, *FAS*, *CASP8*, *CYLD*, *TRIM25*), Cluster 3 had 5 genes (*NLRP1*, *NLRP3*, *NLRP12*, *NOD1*, *IL18*), Cluster 4 consisted of 3 genes (*REL*, *RELB*, *CD40*), Cluster 5 included 2 genes (*PSME1*, *PSME2*), and Cluster 6 contained 2 genes (*VCAM1*, *CXCR4*) (Figure 6).

### 2.8. Quantitative Real-Time PCR (qRT-PCR) Validation

**Figure 7:**
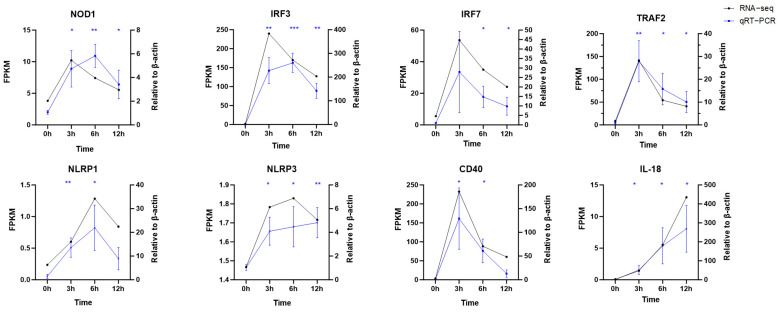
Quantitative real-time PCR (qRT-PCR) validation of 8 immune-related hub genes. The relative expression levels of TRAF2, IRF3, IRF7, NOD1, NLRP1, NLRP3, CD40, and IL-18 were detected at 0 h, 3 h, 6 h, and 12 h post poly I:C stimulation. β-actin was used as the internal reference gene, and the relative expression was calculated using the 2^(−ΔΔCt)^ method. Asterisks denote statistical significance: * *p* < 0.05, ** *p* < 0.01 and *** *p* < 0.001.

Based on KEGG and PPI analyses, 8 immunity-related genes were selected from 5 significantly enriched signaling pathways, including NOD-like receptor, RIG-I-like receptor, TNF, NF-kappa B, and Toll-like receptor signaling pathways, for quantitative PCR verification. The selected genes were TRAF2, IRF3, IRF7, NOD1, NLRP1, NLRP3, IL-18, and CD40. Functionally, TRAF2 participates in multiple pathways, including RIG-I, NF-κB, and TNF signaling; IRF3 and IRF7 are involved in RIG-I-like receptor, NOD-like receptor, and Toll-like receptor pathways; NOD1, NLRP1, NLRP3, and IL-18 are associated with the NOD-like receptor signaling pathway; and CD40 functions in the Toll-like receptor signaling pathway. As shown in Figure 7, expression profiles of the eight DEGs were generally similar to their FPKM obtained in the RNA-seq analysis.

## 3. Discussion

Fish cell lines are good models for studying host anti-disease mechanisms. The brain cell lines of marine fish were established in Japanese flounder (*Paralichthys olivaceus*), humpback grouper (*Cromileptes altivelis*), golden pompano (*Trachinotus ovatus*), and spotted knifejaw [15,16,17]. While the mechanisms of fish immunity are very complex, it is necessary to use high-throughput sequencing and bioinformatics methods for research. To investigate the antiviral immune response of the spotted knifejaw, we established an in vitro model of viral infection by stimulating brain cells with poly (I:C). Transcriptome analysis was constructed with 3, 6, and 12 h post-stimulation compared with 0 h. Thousands of DEGs at each time point reflect the complexity of fish brain cells undergo viral challenge. To reveal the molecular mechanisms, three research strategies were conducted after transcriptome sequencing, and the major signal pathway and hub genes were identified.

### 3.1. Time-Series Expression Pattern Profiles

Time-series expression analysis identified 12 clusters that revealed the dynamic regulatory process of antiviral immune responses in brain cells. This phased response aligns closely with the well-established “recognition-effector-recovery” model of antiviral immunity in mammals [18], suggesting the conservatism of viral immune regulatory strategies between teleosts and higher vertebrates. DEGs in cluster 6 and cluster 10 demonstrated the recognition of host-derived damage-associated molecular patterns (DAMPs) during initial viral invasion, through activity of the RIG-I-like receptor pathway, NOD-like receptors and Jak-STAT signal pathway, which was similar to previous studies in turbot and Japanese flounder [19,20]. Then the DEGs in cytosolic DNA-sensing, RNA degradation, and protein export pathways may target viral RNA replication intermediates at later stages of infection in cell cycles [21].

### 3.2. GO and KEGG Identified Signaling Pathways

In this study, the GO terms of three comparison groups reflect the process of cellular response to poly I:C stimulation, from “response to stimulation” and “regulation of cellular process” to “DNA replication” and “transferase activity”, and then “antigen processing and presentation”. This process was consistent with the fish brain infected with NNV [22].

NOD-like receptors, as intracellular pattern recognition receptors, recognize viral components and trigger downstream inflammatory cascades; RIG-I-like receptor activates type I interferon responses by detecting viral double-stranded RNA, constituting a fundamental antiviral defense mechanism in teleosts [23,24]. These receptors were conserved in spotted knifejaw with other teleosts, suggesting the conserved activation of classical pathways in virus-infected teleosts [25]. Concurrently, KEGG enrichment analysis of co-expressed genes revealed sustained upregulation of key components in these pathways, including TRAF2, IRF3, and NLRP3. This expression pattern was the same as observations in NNV-infected grouper, in which TRAF2 acts as an adaptor linking RIG-I to downstream signaling molecules to initiate interferon responses [26]. Meanwhile, phosphorylation of IRF3/7 represents a critical step driving type I interferon production, which will be studied in our future research [27].

The persistent enrichment of the signal transduction pathway, such as the NF-κB pathway, TNF signal pathway, and p53 signal pathway, suggests their major role in regulating inflammatory mediators, including IL-1β and TNF-α, consistent with antiviral functions in zebrafish [28]. Besides inflammation regulation, the NF-κB pathway also functions as a critical pivot for cell survival. It counteracts programmed cell death induced by various stressors, including oxidative and cytotoxic damage, through upregulation of anti-apoptotic genes such as Bcl-2 family members, thereby maintaining cellular homeostasis [29]. Meanwhile, the significant enrichment pathways, such as the cytosolic DNA-sensing pathway, DNA replication, and mismatch repair, imply that fish brain cells maintain the ability to repair cells under viral mimic stimulation [22].

### 3.3. PPI Analysis Identified Six Clusters of Hub Genes

Through PPI analysis of 1398 common DEGs, six functional clusters comprising 30 hub genes were identified. Cluster 1 (12 genes) is primarily associated with the JAK-STAT and AP-1 signaling pathways, which mediate the transcription of interferons (IFNs) and pro-inflammatory cytokines such as IL-8 and TNF-α, thereby initiating innate immunity as the first line of defense against viral infection. It also participates in the regulation of cell cycle and translation processes, reflecting a dynamic balance between host antiviral responses and viral replication requirements [30]. In fish immunity, the JAK-STAT pathway represents a core mechanism for cytokine-mediated immunoregulation, with STAT1 and STAT3 functioning as signal transducers and transcriptional activators that drive the expression of interferon-stimulated genes (ISGs) [31]. *IRF3*, *IRF7* and *IRF9* serve as key regulators of type I interferon production. Studies in fish species such as zebrafish (*Danio rerio*) and carp (*Cyprinus carpio*) have demonstrated significant upregulation of *IRF7* and *STAT1* upon viral challenge, underscoring their essential roles in antiviral defense [32]. Additionally, *TRAF2* and *TYK2* are involved in Toll-like receptor (TLR) and cytokine receptor signal transduction, further bridging innate and adaptive immune responses [33].

Hub genes in cluster 2 were implicated in the regulation of necroptosis and inflammatory signaling. This cluster mediates CYLD-dependent activation of the RIPK3-MLKL necroptotic pathway, which eliminates intracellular viruses through cytolysis while simultaneously releasing damage-associated molecular patterns (DAMPs) such as ATP and HMGB1. These DAMPs subsequently recruit macrophages and neutrophils into the brain parenchyma, amplifying inflammatory cascades and potentially contributing to the imbalance between tissue injury and host defense during viral infection in fish [34]. Genetically, these genes are associated with ubiquitination modification, apoptosis, and inflammatory responses. Specifically, RNF31 (HOIP) and RBCK1 (HOIL-1) function as core components of the linear ubiquitin chain assembly complex (LUBAC) that facilitates NF-κB signaling activation, while CYLD acts as a deubiquitinating enzyme providing negative feedback regulation of this pathway [35]. In fish immunity, apoptosis pathways mediated by FAS and CASP8 play crucial roles in eliminating infected cells and maintaining immune homeostasis. For example, CASP8 participates in virus-induced apoptoticin in Japanese flounder (*Paralichthys olivaceus*) [36]. Additionally, TRIM25 functions as an E3 ubiquitin ligase in RIG-I-mediated antiviral signaling. Studies have confirmed that fish homologs, such as TRIM25 in medaka (*Oryzias latipes*), activate MAVS signaling via ubiquitination to defend against RNA viral infection [37].

Hub genes in cluster 3 were primarily enriched in the inflammasome activation pathway and NOD-like receptor (NLR) signaling. NLRP3 inflammasome activation facilitates the maturation of IL-1β and IL-18, thereby initiating inflammatory responses. In fish species such as the large yellow croaker (*Larimichthys crocea*), the NLRP3 inflammasome has been demonstrated to play a central role in defense against bacterial infections, including *Vibrio alginolyticus* [38]. As an intracellular pattern recognition receptor, NOD1 recognizes bacterial peptidoglycan and activates the NF-κB pathway, with its immunological functions well established in teleosts such as rainbow trout (*Oncorhynchus mykiss*) [39]. Genes from Cluster 1 and Cluster 3 also exhibited up-regulation in the Toll-like signaling pathway (ko.04620), NOD-like receptor signaling pathway (ko.04621), and RIG-I-like receptor signaling pathway (ko.04622), which were similar to previous research in orange-spotted grouper [24]. Therefore, the function of these hub genes in spotted knifejaw against NNV needs to be studied further.

## 4. Materials and Methods

### 4.1. Cells

Spotted knifejaw brain cell line (OPB) was obtained from Dr. Songlin Chen’s lab and preserved in the Cell Bank of National Marine Genetic Resource Center, Yellow Sea Fishery Research Institute [40]. The access number is YSFRI-C-2020-OPB. After resuscitation, the cells were cultured in L-15 medium supplemented with 15% serum (Gibco, Burlington, ON, Canada) and maintained at 24 °C in a constant-temperature incubator. For every 500 mL of L-15 medium, the following additives were included: 1 mL of basic fibroblast growth factor (bFGF; Invitrogen, Waltham, MA, USA) at a concentration of 5 ng/mL, 14 μL of β-mercaptoethanol (Amresco, Solon, OH, USA), and 2% penicillin-streptomycin-amphotericin B solution (Solarbio, Beijing, China). When OPB reached approximately 80% confluency in the culture flask, subculturing was performed at a 1:2 split ratio using trypsin-EDTA digest solution (Solarbio, Beijing, China).

### 4.2. Poly I:C Stimulation, RNA Extraction, and Transcriptome Sequencing

OPB cells were seeded into 12-well plates at 70–80% confluency and cultured in Leibowitz’s L-15 medium supplemented with 10% fetal bovine serum. After 20 h of incubation, the cells were stimulated with 50 μg/mL Poly I:C for 0, 3, 6, and 12 h. Total RNA was then extracted from each sample using TRIzol reagent according to the manufacturer’s instructions. RNA purity and concentration were assessed using a spectrophotometer, and integrity was evaluated with an Agilent 2100 Bioanalyzer (Agilent Technologies, Santa Clara, CA, USA). The extracted total RNA from OPB cells was subsequently sent to BGI (Qingdao, China) for library construction and transcriptome sequencing.

### 4.3. Quality Control and Alignment of Transcriptome Sequencing Data

Raw sequencing data were processed to obtain high-quality clean reads using SOAPnuke (v1.4.0). Reads were filtered out if they contained adapter sequences, had an unknown base (N) content exceeding 5%, or contained more than 20% of bases with a Phred quality score below 15. The resulting clean reads were stored in FASTQ format. Subsequently, these clean reads were aligned to the *Oplegnathus punctatus* reference genome using HISAT2 (v2.1.0) and to the reference gene sequences using Bowtie2 (v2.2.5).

### 4.4. Screening of Differentially Expressed Genes (DEGs)

DEGs were identified using the DEseq2 software package (v1.50.2). Genes meeting the criteria of an absolute fold change > 2 and an adjusted *p*-value < 0.05 were considered statistically significant. The Pearson correlation between twelve samples was calculated using the cor function in R (v4.5). Using the 0 h group as the control, significant DEGs were separately identified and enumerated for the 3, 6, and 12 h time points.

### 4.5. Time-Series Expression Pattern Analysis

Time-series gene expression patterns were analyzed using the soft clustering algorithm in the Mfuzz package (v2.34.0). This algorithm assigns genes into distinct clusters based on similarities in their expression profiles across the different time points (0, 3, 6, and 12 h post-stimulation). Genes showing consistent expression trends were assigned to the same cluster, enabling the identification of co-regulated gene groups and supporting further analysis of temporal expression dynamics.

### 4.6. Functional Enrichment Analysis of Differentially Expressed Genes

GO and KEGG pathway enrichment analyses were conducted on the significantly DEGs using the Dr. Tom platform of BGI technology (https://biosys.bgi.com/ (accessed on 22 July 2025). Terms or pathways with a corrected Q-value of ≤0.05 were considered statistically significant.

### 4.7. Protein–Protein Interaction (PPI) Network Analysis

To identify common DEGs across different time points, a Venn diagram analysis was performed using the DEGs identified from comparisons of each post-stimulation time point (3, 6, and 12 h) with the 0 h control group. The resulting shared DEGs were further analyzed through KEGG pathway enrichment, following the procedure detailed in Section 4.6.

A protein–protein interaction (PPI) network was constructed for these DEGs using the STRING database (http://www.string-db.org (accessed on 30 July 2025)). To predict functional interactions, protein sequences of the DEGs were mapped to their corresponding orthologs in *Homo sapiens*, the reference species in the STRING database. The resulting interaction dataset was downloaded and imported into the Cytoscape (v3.10.3) software for network visualization and subsequent identification of hub genes.

### 4.8. Validation by Quantitative Real-Time PCR (qRT-PCR)

To validate the transcriptome sequencing results, eight genes (TRAF2, NLRP1, NLRP3, NOD1, IRF3, IRF7, IL-18, and CD40) were selected from the 1398 differentially expressed genes and assessed via qRT-PCR. β-Actin was employed as the internal reference gene. Gene-specific primers were designed using the NCBI BLAST-Primer (version 2.5.0) (www.ncbi.nlm.nih.gov/tools/primer-blast/ (accessed on 30 July 2025)) tool and synthesized by Tsingke Biotechnology Co., Ltd. (Beijing, China).

The qRT-PCR reaction was conducted in a final volume of 10 μL, containing 5 μL of SYBR Green qPCR Master Mix, 0.2 μL each of the forward and reverse primers (10 μM), 1 μL of cDNA template, and 3.6 μL of RNase-free ddH_2_O. The thermal cycling protocol comprised an initial denaturation at 95 °C for 30 s, followed by 40 cycles of denaturation at 95 °C for 5 s and annealing/extension at 60 °C for 30 s. Gene expression levels were quantified using the comparative 2^(−ΔΔCt)^ method, with β-actin as the internal reference. The results were visualized using GraphPad Prism software (v10.1.2) [41].

### 4.9. Statistical Analysis

All quantitative real-time PCR (qRT-PCR) data are presented as the mean ± standard error of the mean (SEM) from three independent biological replicates. Differences in gene expression levels across time points (0 h, 3 h, 6 h, and 12 h post-stimulation) were evaluated for statistical significance using an independent-samples t-test for pairwise comparisons (each time point versus the 0 h control). Data analysis and graph visualization were performed using GraphPad Prism software (v10.1.2). Statistical significance is indicated as follows: * *p* < 0.05, ** *p* < 0.01, and *** *p* < 0.001.

## 5. Conclusions

Using an in vitro brain cell culture system combined with a poly I:C stimulation model and transcriptomic sequencing, this study characterized the DEGs in spotted knifejaw at multiple time points (3, 6, and 12 h) following poly I:C stimulation. Analysis revealed six interactive gene clusters and 30 hub regulatory genes, which were predominantly enriched in innate immune signaling pathways and disease-response pathways. These results provide preliminary insight into the molecular immune mechanisms underlying the anti-NNV response in spotted knifejaw, offering a theoretical foundation for disease-resistant breeding and disease control strategies in marine fish.

## Figures and Tables

**Table 1:** Raw sequencing data and statistics of filtered readout quality of spotted knifejaw brain cell lines.

Sample	Total Raw Reads (M)	Total Clean Reads (M)	Total Clean Bases (Gb)	Clean Reads Q20 (%)	Clean Reads Q30 (%)	Clean Reads Ratio (%)
infected0_1	45.57	43.45	6.52	97.98	93.99	95.34
infected0_2	47.33	42.82	6.42	98.12	94.26	90.48
infected0_3	43.82	41.99	6.3	97.94	93.87	95.82
infected3_1	45.57	43.54	6.53	97.91	93.83	95.55
infected3_2	47.33	42.26	6.34	98.14	94.47	89.3
infected3_3	45.57	43.52	6.53	97.93	93.85	95.5
infected6_1	43.82	42.3	6.35	97.84	93.59	96.54
infected6_2	43.82	42.33	6.35	98.06	94.25	96.59
infected6_3	45.57	43.57	6.54	98.25	94.64	95.6
infected12_1	43.82	42.25	6.34	98.17	94.41	96.42
infected12_2	43.82	42.89	6.43	98	93.81	97.87
infected12_3	45.57	43.25	6.49	98.19	94.42	94.91

## Data Availability

Sequencing data and matrix data generated in this study have been deposited in the CNGB Sequence Archive (CNSA) of China National GeneBank DataBase (CNGBdb) under accession code CNP0008021.

## Supplementary Materials

The following supporting information can be downloaded at: https://www.mdpi.com/article/10.3390/ijms27021101/s1.
