# Supplementary material for: Transcriptome Analysis and Hub Gene Identification in the Brain Cell Lines of the Spotted Knifejaw (Oplegnathus punctatus) After Poly (I:C) Stimulation
Source: Int J Mol Sci. 2026 Jan 22;27(2):1101. doi: 10.3390/ijms27021101 (PMC12842581; doi:10.3390/ijms27021101)
Supplement: Supplementary file 1 [file ijms-27-01101-s001.zip › ijms-4007415-supplementary.pdf]

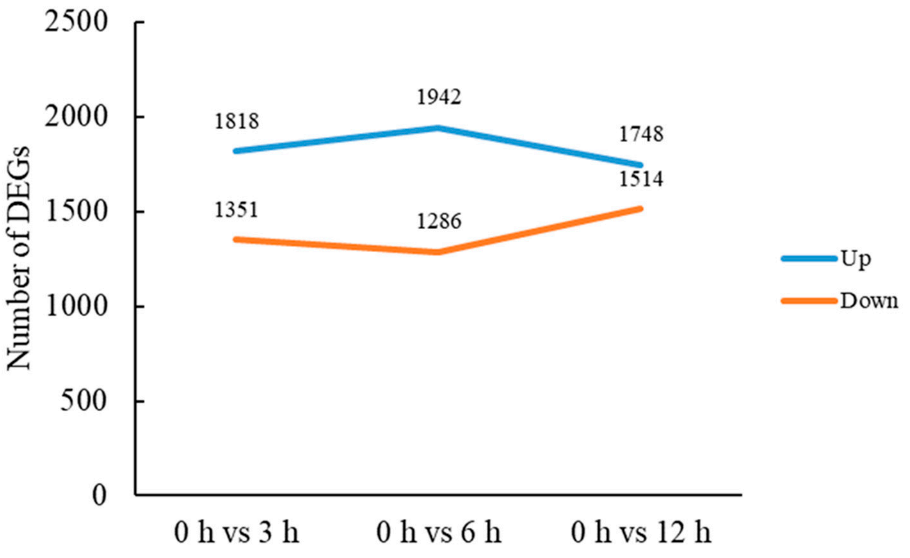

Figure S1. The number of differentially expressed genes in the comparison group at adjacent time points

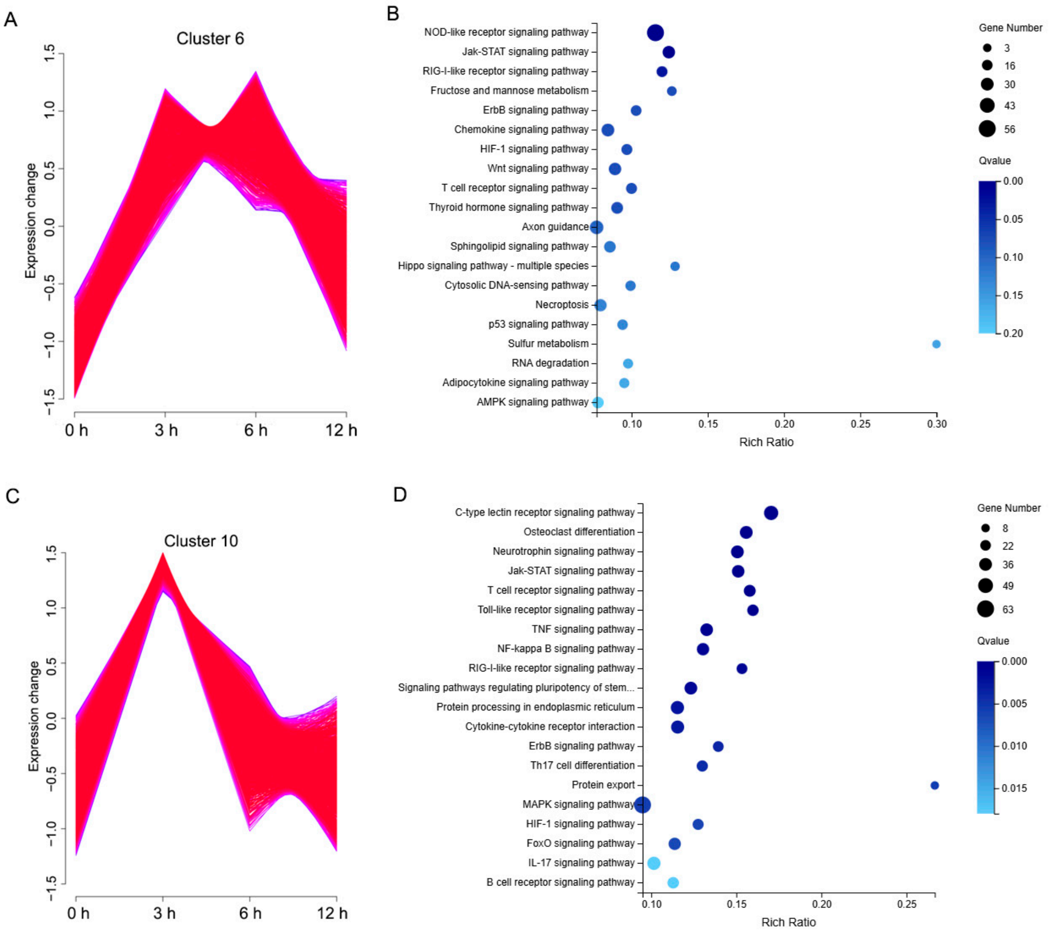

Figure S2. Gene Cluster and KEGG Enrichment Bubble Charts of the cluster6 (A-B) and cluster 10 (C-D).
